# Multicenter Study to Validate a Hospitalization Risk Assessment Tool in Hemodialysis Patients

**DOI:** 10.7759/cureus.51419

**Published:** 2023-12-31

**Authors:** Muhammad Nauman Hashmi, Hammad Raza, Muhammad A Khan, Shazia Rani, Muhammad Naeem Shaikh, Abdulsalam Soomro, Ahmed Elsoul, Ahmed Almokhtar Abdallah, Esraa Ahmed, Maged Ismael, Eman Alharbi, Fayez Hejaili

**Affiliations:** 1 Hemodialysis, Ministry of National Guard Health Affairs, Jeddah, SAU; 2 Medical Education, King Saud Bin Abdulaziz University for Health Sciences, Jeddah, SAU; 3 Research, King Abdullah International Medical Research Center, Jeddah, SAU; 4 Medicine, Ministry of National Guard Health Affairs, Jeddah, SAU; 5 Clinical Dietitian, Ministry of National Guard Health Affairs, Jeddah, SAU; 6 Nephrology, King Abdulaziz Medical City, Riyadh, SAU

**Keywords:** risk assessment tool, mortality, hospitalization, malnutrition, hemodialysis, protein energy wasting (pew)

## Abstract

Introduction

Protein-energy wasting is a prevalent condition in patients with chronic kidney disease. Our goal was to validate the risk assessment tool (Hashmi’s tool) in multiple centers, developed in 2018, as it was easily applicable and cost-effective.

Methods

The following variables were scored as 0, 1, 2, or 3 as per severity: body mass index, HD vintage in years, functional capacity, serum albumin, serum ferritin, and the number of co-morbid conditions (diabetes mellitus, hypertension, ischemic heart disease, and cerebrovascular disease). This scoring system was applied to maintenance hemodialysis patients in six different centers. The patient's record was evaluated for two years. Patients were divided into low-risk (score <6) and high-risk (score ≥6). We compared the two groups using the chi-square test for the difference in hospitalization and mortality.

Results

A total of 868 patients' records were analyzed, and the maximum score was 13 with the application of Hashmi’s tool. Four hundred twenty-nine patients were in the low-risk group, and 439 patients fell into the high-risk group. Four hundred sixty-seven patients were male, and 401 were females; 84% had hypertension, and 54% had diabetes mellitus. In the high-risk group, we identified more females. Patients' likelihood of being in the high-risk group was higher if they had diabetes mellitus, hypertension, or ischemic heart disease. Hospitalization due to vascular or non-vascular etiologies was more common in the high-risk group (p=0.036 and p<0.001, respectively). A total of 123 patients died during the study period, 92 from the high-risk group as compared to 31 from the low-risk group. This was three times higher and statistically significant (p<0.001).

Conclusion

Using a simple and cost-effective tool, we have identified malnourished patients who are at risk of hospitalization and mortality. This study has validated the previous work at a single center, which has now been reflected in six dialysis units across Saudi Arabia.

## Introduction

Protein-energy wasting (PEW) is a significant and prevalent condition in kidney disease patients [1]. PEW is a complex syndrome due to metabolic changes resulting from uremia, poor functional capacity, increased risk of infections, frequent hospitalization, and an increased risk of mortality. PEW is the state of decreased body stores of protein and energy fuels. Causes include nonspecific inflammatory processes; transient, intercurrent catabolic illnesses; nutrient losses into dialysate; academia; and endocrine disorders. PEW increases the risk of infection, risk of hospitalization, and risk of mortality. The prevalence of PEW in the hemodialysis population was reported to be 26% in a cross-sectional study [2]. A meta-analysis of observational studies showed 28-54% of dialysis patients had PEW [3].

A few nutritional assessment tools have been used to identify patients with malnutrition. Subjective global assessment (SGA) is generally used; it consists of medical history and examinations [4]. The malnutrition-inflammation score (MIS) combines SGA with laboratory parameters, body mass index, and hemodialysis vintage to assess nutritional deficiency [5]. The geriatric nutritional risk index (GNRI), malnutrition universal screening tool (MUST), mini-nutritional assessment short form (MNA-SF), and nutritional risk index for Japanese hemodialysis patients (NRI-JH) are also used to assess the risk of malnutrition [6]. Recently, the Global Leadership Initiative on Malnutrition (GLIM) has established a two-step method for malnutrition identification: first to screen and identify at-risk patients and second to diagnose and grade the severity of malnutrition [7].

The above-mentioned screening tools help in the identification of patients at risk of malnutrition, and the results showed a high risk of all-cause mortality in the majority of published studies. These tools use a blend of subjective and objective criteria and assign scores according to severity. Hashmi et al. [8] published a simplified and cost-effective objective tool in 2018 that included six parameters and scored them according to severity. The results were divided into low-risk (score <6) and high-risk (score ≥6) groups. Those who were at high risk were at a greater risk of hospitalization and mortality. This study was conducted at a single center. The group suggested performing a larger-scale study; hence, a multicenter trial was designed to validate Hashmi’s scoring tool.

## Materials and methods

Six variables, namely, body mass index, functional capacity, HD vintage in years, serum albumin, serum ferritin, and co-existing comorbid conditions (diabetes mellitus, hypertension, ischemic heart disease, and cerebrovascular disease), were scored according to severity as 0, 1, 2, or 3. Functional capacity was assessed based on patient or family reports. Occasional difficulty in daily life activities was defined as less than three times per week. Difficulty in independent activities was defined as the inability to use the toilet unaided, eat independently, and self-dress.

This tool was applied to six hemodialysis units under the Ministry of National Guard Health Affairs across Saudi Arabia. Regular patients on three times per week dialysis with age 16 years and above were included in this study. Transient/tourist patients, aged less than 16 years, who visited twice per week, were excluded from the study. Patients' data were recorded from the electronic system of the hospital (best care) and retrospectively analyzed from January 2021 to December 2022 (two-year timeframe). Laboratory parameters were recorded every quarter. Verbal Informed consent was obtained from all patients.

Institutional Review Board (IRB) approval was taken in December 2022. After approval (IRB/2784/22) data collection was done from all six hemodialysis centers of the Ministry of National Guard Health Affairs and compiled in an Excel spreadsheet. The scoring tool was applied. Patients were divided into low-risk (score <6) or high-risk (score ≥6) groups. These two groups were compared for mortality and hospitalization rates.

## Results

Hashmi’s tool (sum of BMI, functional capacity, number of years on HD, serum albumin, serum ferritin, and comorbidities) was used. These variables were given a score of 0, 1, 2, or 3, as per the categorization in Table 1. This scoring tool was applied quarterly, and the scores were recorded. A total of 1,129 patients from six dialysis units under the Ministry of the National Guard Health Affairs were reviewed. After applying the inclusion/exclusion criteria, 261 patients were excluded. Data were reviewed from January 2021 to December 2022. Patient distribution from six centers (Riyadh North, 228; Riyadh South, 190; Jeddah, 200; Madinah, 98; Makkah, 91; and Hail Centre, 61).

**Table 1:** Hashmi's scoring tool Co-morbidities (hypertension, diabetes mellitus, ischemic heart disease, and cerebrovascular disease)

	0	1	2	3
BMI	≥20	18-19.9	16-17.9	<16
Functional capacity	Normal	Occasional difficulty in daily activities	Difficulty in independent activities	Bed/chair ridden
No of years on HD	< 1 year	1- <3 years	3-4 years	> 4 years
Serum albumin	≥35 g/L	32-35 g/L	28-31 g/L	<28 g/L
Serum ferritin	<700 μg/L	701-800 μg/L	801-1,000 μg/L	>1,000 μg/L
Co-morbidities	1	2	3	>3

From 868 patients’ data, analysis showed a minimum recorded age of 19 years and a maximum of 103 years (mean of 59.6 years ± SD 15.57), mean hemoglobin of 113.6 g/l ± SD 11.99, and K t/v mean ± standard deviation of 1.7 ± 0.49. More than 90% (n=786) of patients had a BMI of >20, 88.4% (n=767) had serum albumin of >35 g/L, 72% (n=628) had been on hemodialysis for >4 years, and 77% (n=669) had serum ferritin of <700 mcg/l. Functional capacity was variable, with 38% (n=331) being normal, 22.6% (n=196) having occasional difficulty, 19% (n=165) having difficulty in independent activities, and 20.3% (n=176) of patients being chair- or bed-bound. Among the co-morbid conditions, 21.8% (n=189) had one illness, 32% (n=278) had two, 29.7% (n=258) had three, and 16.5% (n=143) had more than three co-morbidities.

With the help of Hashmi’s scoring tool, patients were categorized into low-risk (score <6) and high-risk groups (score ≥6). Our data showed a minimum recorded score of 1 and a maximum score of 13. Of the total, 429 patients were in the low-risk category and 439 in the high-risk category. A comparison of the demographic data of both groups is shown in Table 2. In this table, the association of patient characteristics with the score was assessed using the chi-squared test. Females were more frequent in the high-risk group (233, 58.1%) than in the low-risk group (168, 41.9%), which was statistically significant (OR: 1.76; 95% CI: 1.34-2.30; p<0.001). Divorced or widowed participants were more likely to have a higher risk score compared with singles in the low-risk group, which was significant (p=0.001), and married participants had almost the same distribution. Patients who were non-diabetics (OR: 5.28; 95% CI: 3.95-7.06; p<0.001) or non-hypertensive (OR: 2.16; 95% CI: 1.48-3.16; p<0.001) were found to have a low-risk score, while those with these diseases had a high-risk score with a significant p-value. Among patients with ischemic heart disease, the disease placed them in the high-risk category, which was also significant (OR: 4.38; 95% CI: 3.56-6.55; p<0.001). Patients on erythropoietin had no significant difference in being in the low-risk or high-risk group (p>0.05).

**Table 2:** Low-risk vs high-risk patients' demographics and clinical data Data are presented in N (numbers), and the percentages are shown next to it. The p value is significant at <0.05.

		Hashmi’s Score							
		Low Risk (Score <6)		High Risk (Score ≥6)		OR	95% CI		p
		n=429	%	n=439	%				
Gender									
Male		261	(55.9)	206	(44.1)	1.757	1.34	2.30	<0.001
Female		168	(41.9)	233	(58.1)				
Marital Status									
Married		339	(49.9)	341	(50.1)	Not applicable			<0.001
Single		75	(75.0)	25	(25.0)				
Divorce/Widow		15	(17.0)	73	(83.0)				
Diabetes									
No		282	(70.7)	117	(29.3)	5.280	3.95	7.06	<0.001
Yes		147	(31.3)	322	(68.7)				
Hypertension									
No		90	(65.2)	48	(34.8)	2.163	1.48	3.16	<0.001
Yes		339	(46.4)	391	(53.6)				
Ischemic Heart Disease									
No		347	(62.9)	205	(37.1)	4.830	3.56	6.55	<0.001
Yes		82	(25.9)	234	(74.1)				
Patient on Epo									
No		105	(50.5)	103	(49.5)	1.057	0.77	1.44	0.727
Yes		324	(49.1)	336	(50.9)				

Patients' hospitalization due to vascular causes (OR: 1.33; 95% CI: 1.02-1.75; p=0.036), non-vascular causes (OR: 2.61; 95% CI: 1.98-3.45; p<0.001), and mortality (OR: 3.39; 95% CI: 2.20-5.22; p<0.001) were significantly higher in the high-risk group than in the low-risk group. We noted that 31 patients in the low-risk group died during the study period, and 92 died in the high-risk group. This was three times higher and statistically significant (Table 3).

**Table 3:** Hospitalisation and mortality in low-risk and high-risk groups Data are shown in percentages. The p value is significant at <0.05.

Hashmi’s Score						
	Low Risk	High Risk	OR	95% CI		p
With Vascular Admission	45.3%	54.7%	1.335	1.02	1.75	0.036
Non-vascular Admission	39.7%	60.3%	2.614	1.98	3.45	<0.001
Mortality Observed Period°	25.2%	74.8%	3.388	2.20	5.22	<0.001

## Discussion

PEW is a significant multifactorial issue, especially in patients with end-stage kidney disease. Volume overload, oxidative stress, poor nutritional intake, hormonal dysregulation, metabolic acidosis, raised inflammatory cytokine levels, and dialysis treatment-related factors were the main contributing factors. This leads to PEW, resulting in several parameter derangements: a drop in body mass index, low albumin and lipid profile, raised inflammatory markers, increased cardiovascular mortality, and an overall increase in morbidity and mortality [9].

Nutritional risk assessment tools have been recommended as practical tools for identifying malnutrition in patients. The GLIM recommends screening patients and grading them according to severity [10].

Hashmi et al. [8] proposed a more objective tool for assessing malnourished patients. A comprehensive scoring system is easy to apply and cost-effective and helps stratify patients.

In our study, we identified more female patients in the high-risk group who are at elevated risk of hospitalization and mortality. This inclination has been proven in other studies. A cross-sectional study from Spain in advanced chronic kidney disease patients showed a prevalence of 30.1% of PEW, with a significant difference between men and women (22.8 vs. 33.8%, p<0.005) [11]. Another single-center study from Jeddah showed female predominance in the malnourished group [12]. A similar finding was observed in a hemodialysis patient population from Nepal [13].

Co-morbidities such as diabetes mellitus and ischemic heart disease have a direct effect on quality of life and put patients at further risk of malnutrition. A study from Japan showed more sarcopenia in the dialysis population in comparison with non-dialysis patients. They further reported that the presence of diabetes mellitus induces malnutrition [14]. A similar observation from a study in 2019 found that the presence of diabetes mellitus was an independent contributor to sarcopenia [15]. Oxidative stress is a major contributor to endothelial injury and escalates atherosclerosis [16]. Patients with a higher risk of malnutrition have a higher probability of cardiovascular disease, which is reported to be the leading cause of mortality in dialysis patients [17].

Marital status has a greater impact on the risk of malnutrition, especially in widowed or divorced, as compared to being married or living with a family. A systematic review and meta-analysis of observational studies state that, by tackling socioeconomic situations, the risk of malnutrition can be decreased. Supporting the patients who live alone, widowed, or divorced [18].

Hemodialysis patients require frequent hospitalization, which could be because of vascular access dysfunction or other non-vascular causes [19]. We identified more hospitalizations with vascular (54.7%) and non-vascular (60.3%) causes in the high-risk group. Patients in both categories (vascular and non-vascular) with high scores required frequent hospitalization. This signifies that patients who are at risk of malnutrition require frequent hospital admissions. This has a major impact on quality of life and signifies health budget expenditures.

During our study period, 123 patients died, 31 from the low-risk group and 92 from the high-risk category. This is three times more in the high-risk group. Studies across the globe have reported a high risk of mortality in malnourished patients with advanced kidney disease [20]. This shows that patients who are at risk of malnourishment should be identified and managed in order to decrease morbidity and mortality. It needs a holistic, multidisciplinary approach. Applying the tool proposed by Hashmi et al., we are proposing a simplified tool that identifies patients at risk of hospitalization and mortality. This multicenter trial validates the scoring tool in the population studied and helps in concentrating on these patients in order to minimize morbidity and mortality. These patients require close monitoring with vigorous evaluation and management.

The sample size in this study is sufficient to suggest the application of this tool in hemodialysis centers. This does not involve any additional cost, as the parameters mentioned are regularly done in hemodialysis centers. The application of the tool is quarterly-based, so no additional workforce is required, and we can easily add it to regular clinical activities.

## Conclusions

This study validates previously described Hashmi's tool in identifying hemodialysis patients at risk of hospitalization and mortality in multiple centers. This tool is easy to apply, does not require extra cost, and can be part of regular hemodialysis unit rounds. This will help identify high-risk patients. This group can be monitored closely and can be a multidisciplinary approach to reduce morbidity and mortality.

This study was conducted in one country, so we suggest applying this tool in different geographical areas in order to further evaluate and validate this scoring tool. Further research using this tool in order to monitor therapeutic decisions and for guidance to reduce morbidity and mortality is warranted.
